# The role and function of HDL in patients with diabetes mellitus and the related cardiovascular risk

**DOI:** 10.1186/s12944-017-0594-3

**Published:** 2017-10-30

**Authors:** Marek Femlak, Anna Gluba-Brzózka, Aleksandra Ciałkowska-Rysz, Jacek Rysz

**Affiliations:** 1 105 Military Hospital with Outpatient Clinic in Żary, Domańskiego 2, 68-200 Żary, Poland; 2 Department of Nephrology, Hypertension and Family Medicine, WAM Teaching Hospital of Lodz, Żeromskiego 113, Łódź, 90-549 Poland; 3 Department of Oncology, Palliative Care Ward, Medical University of Lodz, Łódź, Poland; 4 Department of Nephrology Hypertension and Family Medicine, Medical University of Lodz, Żeromskiego 113, Łódź, 90-549 Poland

**Keywords:** Diabetes mellitus, HDL cholesterol, Subfractions, Cardiovascular risk

## Abstract

**Background:**

Diabetes mellitus (DM) is a major public health problem which prevalence is constantly raising, particularly in low- and middle-income countries. Both diabetes mellitus types (DMT1 and DMT2) are associated with high risk of developing chronic complications, such as retinopathy, nephropathy, neuropathy, endothelial dysfunction, and atherosclerosis.

**Methods:**

This is a review of available articles concerning HDL subfractions profile in diabetes mellitus and the related cardiovascular risk. In this review, HDL dysfunction in diabetes, the impact of HDL alterations on the risk diabetes development as well as the association between disturbed HDL particle in DM and cardiovascular risk is discussed.

**Results:**

Changes in the amount of circulation lipids, including triglycerides and LDL cholesterol as well as the HDL are frequent also in the course of DMT1 and DMT2. In normal state HDL exerts various antiatherogenic properties, including reverse cholesterol transport, antioxidative and anti-inflammatory capacities. However, it has been suggested that in pathological state HDL becomes “dysfunctional” which means that relative composition of lipids and proteins in HDL, as well as enzymatic activities associated to HDL, such as paraoxonase 1 (PON1) and lipoprotein-associated phospholipase 11 (Lp-PLA2) are altered. HDL properties are compromised in patients with diabetes mellitus (DM), due to oxidative modification and glycation of the HDL protein as well as the transformation of the HDL proteome into a proinflammatory protein. Numerous studies confirm that the ability of HDL to suppress inflammatory signals is significantly reduced in this group of patients. However, the exact underlying mechanisms remains to be unravelled in vivo.

**Conclusions:**

The understanding of pathological mechanisms underlying HDL dysfunction may enable the development of therapies targeted at specific subpopulations and focusing at the diminishing of cardiovascular risk.

## Background

Diabetes mellitus (DM) is a major public health problem which prevalence is constantly raising, particularly in low- and middle-income countries [1]. In 2012, total burden of deaths worldwide from high blood glucose was estimated to amount to 3.7 million. 1.5 million deaths were associated with the presence of diabetes, while additional 2.2 million deaths was the result of DM-related increased risks of cardiovascular and other diseases [1]. In 2014, 422 million people in the world suffered from diabetes [1]. Both diabetes mellitus types (DMT1 and DMT2) are associated with high risk of developing chronic complications, such as retinopathy, nephropathy, neuropathy, endothelial dysfunction, and atherosclerosis [2]. According to studies, adult patients with type 1 diabetes (DMT1) poses a less atherogenic fasting lipid profile than people without diabetes, however, the incidence of cardiovascular diseases (CAD) is paradoxically high in this group of patients [3–5]. Even diabetic women were shown to develop CAD earlier than non-diabetic women and they have CAD rates approaching those of men with DMT1 [6, 7]. Changes in the amount of circulating lipids, including the increase in triglycerides and LDL cholesterol as well as the decrease in HDL are frequent also in the course of DMT2 [8]. However, it has been found that dyslipidaemia may precede DM by several years [9].

Numerous studies have shown that HDL cholesterol is strongly and inversely associated with the occurrence of cardiovascular events [10–14]. HDL cholesterol participates in the efflux of cholesterol efflux from peripheral cells as well as in reverse cholesterol transport from these cells to the liver [8]. According to studies, HDL has antioxidative and anti-inflammatory properties. It reduces LDL oxidation [15], inhibits oxidized LDL-induced MCP-1 (monocyte chemoattractant protein 1) production and monocyte transmigration in a co-culture of human aortic endothelial cells and human aortic smooth muscle cells [16, 17] and blunts inflammatory response of endothelial cells to TNF-α (tumour necrosis factor-1) and IL-1 (interleukin 1) stimuli [18]. Finally, it has been demonstrated to exert anti-thrombotic and anti-apoptotic effects [19]. It has been shown that its biological activity may change in various pathophysiological states. In the past it was believed that high level of HDL protects against the occurrence of cardiovascular disease, however new evidence suggest that in some pathological conditions cholesterol HDL may lose its protective properties and become pro-atherogenic. The results of studies confirm that high levels of HDL cholesterol may not be always beneficial. Systematic review and meta-regression analysis of randomized controlled trials testing lipid modifying interventions provided evidence that increasing circulating HDL-C did not reduce coronary heart disease morbidity or mortality [20]. Also The Initiating Dialysis Early and Late (IDEAL) study, in which the efficacy of high to moderate dose statin regimen for the secondary prevention of cardiovascular disease was compared, and The European Prospective Investigation of Cancer, Norfolk (EPIC-Norfolk) [21] demonstrated that highly elevated HDL-C concentrations did not protect against cardiovascular disease.

HDL becomes “dysfunctional” inter alia in type 2 diabetes [5], which may mean that also in pathological state relative composition of lipids and proteins in HDL, as well as enzymatic activities associated to HDL, such as paraoxonase 1 (PON1) and lipoprotein-associated phospholipase 11 (Lp-PLA2), are altered [22]. It has been suggested that plasma HDL cholesterol is not homogeneous, it comprises different particles varying in size, density, apolipoprotein composition, and lipid content. The hypothesis of dysfunctional HDL-C in relation to its activity and reverse cholesterol transport, has been put forward in type 1 diabetes [23]. Generally increased HDL-C does not translate into lower cardiovascular risk in DMT1 patients, but rather an inverse association was observed. However, the exact mechanisms of such relationship remain not fully elucidated. It has been suggested that different HDL subfractions relate to coronary artery disease (CAD) incidence in a different manner.

In this review, HDL dysfunction in diabetes, the impact of HDL alterations on the risk diabetes development as well as the association between disturbed HDL particle in DM and cardiovascular risk is discussed.

### Types of HDL particles obtained using various methods of separation

Numerous studies indicated that HDL particles are highly heterogeneous in size, shape, density and properties. Abundance of different methodologies used to analyse HDL subclasses resulted in the generation of numerous classifications with do not relate with each other. It is generally believed that at the beginning of its formation, HDL is a discoidal and lipid poor [24]. Then, Apo A-I acquires cholesterol and phospholipids via its interaction with the ATP-binding cassette 10 (ABCA1) leading to the formation of pre-β1 HDL particles [24]. These particles gradually accumulate more and more cholesterol. Following the esterification by the enzyme lecithin-cholesterol acyltransferase (LCAT) cholesterol is transferred to the core of HDL particle, forming larger, spherical, α-mobility HDL particles, which may undergo clearance by the hepatic scavenger receptor [24]. Cholesteryl esters can be transferred to VLDL/LDL for catabolism via cholesteryl ester transfer protein (CETP) enzyme.

HDL can be divided into various subclasses, according to its density, size, electrophoretic mobility and apolipoprotein cargo. The use of ultracentrifugation allows for the separation of HDL2 (1.063–1.125 g/mL) and HDL3 (1.125–1.21 g/mL), while gradient gel electrophoresis allows to receive HDL2b (9.7–12.0 nm), HDL2a (8.8–9.7 nm), HDL3a (8.2–8.8 nm), HDL3b (7.8–8.2 nm) and HDL3c (7.2–7.8 nm) or HDL1-HDL10 (Lipoprint) [25]. Also. the obtaining of large HDL, medium HDL, small HDL, spherical, discoidal HDL and many others is possible using various methods.

Proteins, which constitute important part of HDL particles are responsible for their structure and function. Among the most important components of HDL cholesterol there are apolipoproteins (e.g. ApoA-I, ApoA-II, ApoE, ApoJ), enzymes (e.g. LCAT, PON1; LpPLA2’ PAF-AH, GSPx-3), lipid transfer proteins (e.g. PLTP, CETP), acute-phase response proteins (SAA1, SAA4, alpha-2-HS-glycoprotein), complement components and proteinase inhibitors (alpha-1-antitrypsin) and many other [25].

### HDL levels and diabetes risk

Recent studies have indicated that cholesterol HDL may directly alter glucose metabolism [26, 27]. Indeed, HDL cholesterol promotes pancreatic β-cell insulin secretion and modifies glucose uptake in skeletal muscle as shown in different experimental and human settings [27–30]. Therefore, low levels of HDL cholesterol has been suggested to be associated with higher risk of type 2 diabetes in epidemiological studies [31, 32]. Moreover, plasma HDL level increase has been proposed as a therapeutic measure to reduce the risk of type 2 diabetes [33–35]. However, the results of genetic studies evaluating the relationship between HDL cholesterol levels and glycaemic control and risk of type 2 diabetes are conflicting [36–38]. Some studies demonstrated the relationship between HDL particles and lower risk of type 2 diabetes [22, 39, 40]. Hwang et al. [10] found an inverse association between total cholesterol and HDL2 and future development of type 2 DM and this relationship was independent of well-established risk factors for type 2 diabetes. However, they failed to find any correlation between HDL3 cholesterol and future diabetes risk. Also Tabara et al. [41] suggested that high-density lipoprotein (HDL) may exert an antidiabetes function. In their study HDL2 cholesterol levels were inversely associated with HOMA-IR (β = −0.169, *p* < 0.001) and type 2 diabetes (OR = 0.96, *p* = 0.001 Opposite relationship was observed in case of HDL3-C and HOMA-IR (β = 0.054, *p* < 0.001) and type 2 diabetes (OR = 1.04, *p* = 0.181). In turn, a longitudinal analysis demonstrated inverse relationship between HDL2-C and the exacerbation of insulin resistance (β = −0.163, *p* < 0.001) and the inverse risk of type 2 diabetes incidence (odds ratio = 0.98, *p* = 0.006) [41].

It has been proposed that the deletion within ABCA1 may be associated with cholesterol accumulation within cell membrane of beta cells and further results in the hampering of the exocytosis of insulin from secretory granules, and inhibition of insulin secretion [42, 43]. The mutation R230C in ABCA-1, which is associated with reduced cholesterol efflux capacities, was demonstrated to be more frequent in young persons with DMT2 [44]. Animal studies provided the explanation of this phenomenon. It seems that cholesterol accumulation in islet β-cells is the responsible for the pathology.

Moreover, beneficial, apoptosis-inhibiting effects of HDLs on beta cells have also been demonstrated [45]. Fryirs et al. [46] revealed that the incubation of Min6 cells and primary islets with HDLs isolated from human plasma or a constituent of discoidal reconstituted HDLs (rHDLs) or apolipoprotein (apo) A-I or apoA-II enhanced insulin secretion up to 5-fold in a calcium-dependent as well as time and concentration dependent manner [46]. The observation that intravenous reconstituted HDL (rHDL) reduced plasma glucose in patients with type 2 diabetes mellitus by increasing plasma insulin and stimulating AMP-activated protein kinase in skeletal muscle further supports the view that HDLs have the capacity to improve diabetic control and probably postpone the development of new diabetes via several mechanisms [27]. According to Han et al. [30] study, apo A-I was able to stimulate the phosphorylation of the key metabolic regulatory enzyme AMPK and increased glucose uptake in C2C12 myocytes. In turn, Rapizzi et al. [47] reported that HDL-associated sphingolipid S1P could enhance glucose uptake in skeletal muscle through transactivation of the insulin receptor. HDL was reported to reverse the deleterious effects of oxidized LDL on insulin secretion [48, 49]. Recently, it has been shown that HDL can reciprocally increase adiponectin expression in a PI3K-dependent way, which offers a novel indirect way of glucose homeostasis regulation [50]. The treatment with the apo A-I mimetic peptide L-4F increased serum adiponectin levels and decreased IL-1β and IL-6 levels in obese mice and this was accompanied by increased the presence of insulin-sensitive adipocytes [51], improved insulin sensitivity and improved glucose tolerance [52]. Van Linthout et al. [53] reported a decrease in cardiac glycogen content following apo A-I gene transfer in an experimental model of diabetic cardiopathy. They suggested that this effect may be associated with Akt-glycogen synthase kinase (GSK)-3β dependent pathway.

However, the recent Haase et al. [28] study demonstrated that lifelong low levels of HDL cholesterol due to genetic variation in HDL cholesterol-related genes were not associated with increased risk of type 2 diabetes in the general population. They also suggested that low levels of HDL cholesterol per se do not cause type 2 diabetes and but they may be explained by reverse causation, due to a state of prediabetes prior to the diabetes diagnosis. The results of large Schou et al. [38] study also failed to find any association between loss-of-function mutations in ABCA1 and ABCG1 and risk of type 2 diabetes in 40,000 individuals. No relationship between HDL cholesterol related genes and type 2 diabetes has also been reported in recent genome-wide association studies and meta-analyses [54, 55]. Contrary to the aforementioned results, smaller studies of genetic variation in ABCA1 (ATP-binding cassette transporter 1) demonstrated that R230C variant was associated with increased risk of type 2 diabetes [56], while loss-of-function mutations in ABCA1 were proposed to correlate with impaired β-cell function, but not with development of type 2 diabetes [57]. Also, Mackey et al. [58] study demonstrated that decreases in large HDL particles adjusted for confounders were significantly associated with the incidence of diabetes.

### Influence of DM on HDL composition and level

The direct impact of insulin resistance on lipid metabolism in type 2 diabetes DMT2 is quite well-known, while in DM type 1 the mechanisms related to insulin deficiency and dyslipidaemia remain poorly understood and controversial. According to studies, diabetes generally promotes not only quantitative changes in the amount of circulating lipids – particularly an increase in triglycerides and LDL as well as a reduction in HDL but also qualitative and kinetic in nature [59–61]. Decreased plasma concentration, triacylglycerol enrichment, reduced phospholipids, ApoE and ApoM, glycation and increased HDL catabolism are the main changes occurring in diabetes [19]. Altered HDL composition in patients with diabetes results in diminished ability to promote reverse cholesterol transport. Impaired cholesterol efflux from adipose and hepatic cells is mainly related to increased triglyceride and decreased cholesterol content in HDL [62].

Miettinen et al. [63] demonstrated increased markers of cholesterol absorption and decreased markers of cholesterol synthesis in patients with DM type 1 in comparison to control subjects, suggesting that the occurrence of high cholesterol absorption and low cholesterol synthesis in this group of patients with type 1 diabetes. The relationship between gender and lipid levels in patients with DMT1 was analysed by Maahs et al. [64]. They observed that male type 1 diabetic subjects showed higher content of large and lower content of small HDL-C particles than non-diabetic subjects, while DMT1 women had smaller amount of large and higher amount of small dense LDL lipoproteins and reduced LDL size [64]. However, it remains unclear whether the aforementioned lipid abnormalities are due to impaired lipid metabolism associated with DMT1 rather than with glucose control, gender, insulin resistance, and non-regular lifestyle of these patients, or by all these factors in combination. The study of 127 patients with DMT1 demonstrated higher levels of total HDL-C and the lowest density HDL subfraction, apolipoprotein A-I, LPL activity, and adiponectin levels in comparison to control subjects (*P* < .05) [65]. Moreover, Calderon et al. found a relationship between adiponectin and LPL activity and total HDL and its lowest density subfraction.

DM type 2 is associated with dyslipidaemia which involves abnormalities in all types of lipoproteins [19, 66, 67]. According to studies, concentrations of HDL cholesterol are diminished in patients with diabetes mellitus type 2 [68, 69]. Moreover, the predominance of small dense HDL particles which undergo rapid catabolism has been also reported [8, 68, 69].

Hypertriglyceridemia which appears in the course of T2 diabetes mellitus is associated with insulin resistance, hyperglycaemia and hyperinsulinemia. Insulin resistance was shown to increase free fatty acids availability, while hyperinsulinemia and hyperglycaemia promote triglyceride synthesis via the activation of carbohydrate-responsive element-binding protein (ChREBP) [65] and sterol regulatory element-binding transcription factor 1 (SREBF1c) [66], respectively and the consequent increase in cholesteryl ester transfer protein (CETP) activation. Enhanced CETP activity is associated with the enrichment of HDL particles with triglycerides, which is responsible for increased HDL catabolism. Hepatic lipase (HL), which expression and activity is augmented in the presence of hyperglycaemia and insulin resistance, metabolizes triglyceride-rich HDL leading at first to the formation of small HDL particles and then to their accelerated clearance [70, 71]. Therefore, the amount of circulating smaller HDL particles (HDL3) is increased, while the number of large HDL particles (HDL2) is diminished [72]. Also, the content of cholesteryl esters is diminished in HDL particles of DM patients [73]. The alteration of HDL particle lipid composition results in Apo A-I destabilization and its shedding form HDL during lipolysis [19, 74]. The decrease in HDL cholesterol levels may be also associated with the lowering of plasma adiponectin levels in patients with insulin resistance and type 2 diabetes. Vergès et al. [75] demonstrated a negative correlation between HDL-ApoA-I catabolism rate and plasma levels of adiponectin, which was independent of abdominal obesity, insulin sensitivity, age, and sex and plasma lipids. Their finding suggests a direct impact of adiponectin on HDL metabolism, however, the exact mechanism has not been unraveled. Moreover, in patients with type 2 DM decreased plasma concentrations of campesterol and increased levels of lathosterol were observed which mirrors reduced cholesterol absorption and enhanced cholesterol synthesis [76, 77]. Alteration in HDL function and structure are also associated with glycation and oxidation of HDL-associated proteins, changes in gene expression and activity of HDL-metabolizing enzymes. Prolonged inflammation present in DM promotes changes in HDL proteome. All these changes results in the loss of HDL of its normal function and in its “transformation” into a proatherogenic particle. In vitro studies have revealed that HDL glycation is accompanied by the oxidation of HDL lipids and results in diminished cholesterol efflux and reduced HDL affinity binding to fibroblasts. [8, 78–81]. Hyperglycaemia and glycation contribute to disturbed cholesterol efflux, reduced expression of ABCA-1 [82] and scavenger receptor class B type I (SR-BI) [83] and lower HDL antioxidative capacity. Moreover, glycation has been shown to decrease paraoxonase 1 (PON1) activity and to inactivate LCAT. In vitro glycation of HDL was also shown to hamper HDL ability to suppress TNF-α and IL-1β production by lipopolysaccharide-stimulated macrophages [84] as well as to reduce monocyte adhesion to human aortic endothelial cells induced by oxidized LDL [85, 86]. Nobécourt et al. [87] demonstrated that non-enzymatic glycation impaired the anti-inflammatory properties of apolipoprotein A-I and therefore it exerted deleterious effects on HDL key functions. The presence of advanced glycation end products (AGEs) induces changes in the conformation and surface charge of HDL apolipoproteins and decreases the activity of HDL-bound enzymes, including lecithin-cholesterol acyltransferase and paraoxonase-1 [86–88]. However, due to the fact that AGE levels in HDL after in vitro glycation were 5- to 150-fold higher than after in vivo glycation in T2D subjects [86, 87, 89] it is difficult to confirm that such glycation of HDL exerts a direct effect on its anti-inflammatory effects in DMT2.

Apart from glycation, also oxidative modifications of HDL are associated with disturbed HDL function. Negative correlation between HDL oxidation and ABCA1-dependent cholesterol efflux was observed by Zeng et al. [90] who suggested that apolipoprotein A-I was a selective target for myeloperoxidase-catalysed oxidation and its functional impairment was frequent in subjects with cardiovascular disease. Other studies have indicated that oxidative modifications of Apo-AI affect two amino acids (Tyr-166 and Met-148) which are placed within lecithin–cholesterol acyltransferase binding site thus preventing LCAT binding and abolishing its activity [91, 92]. Also paraoxonase 1 is sensitive to oxidation and it becomes inactive following HDL oxidation [93]. Diabetes is associated with the presence of prolonged inflammation. According to studies, inflammation changes HDL proteome converting it from an antiatherogenic particle to a raft of immunological proteins [8]. During acute phase response, Apo A-I content was observed to be diminished due to its replacement by acute phase protein - serum amyloid A [94]. Moreover, the activity of HDL antioxidant enzymes, including PON1, PAF-AH and LCAT, is also reduced during an acute phase response [94]. It has been recently hypothesized that the relative distribution of Lp-PLA2 between LDL and HDL determines whether it exerts pro- or anti-inflammatory effects - Lp-PLA2 in HDL is anti-inflammatory while Lp-PLA2 associated to apoB-containing lipoproteins is pro-inflammatory [95].

The presence of chronic inflammatory state promotes the transformation of HDL into proinflammatory particle which no longer mitigates inflammatory response stimulated by oxidized LDL, but it aggravates the situation [96]. Moreover, Chiba et al. suggested that HDL proteasome change by the inflammation results in HDL ability to bind components of the extracellular matrix, such as vascular proteoglycans. Also Dullaart et al. [97] demonstrated reduced PON-1 activity, HDL cholesterol and apoA-I in T2DM (all *p* < 0.05). Despite the lack of HDL particle concentration change, their distribution was found to be different. Large & medium HDL particles, and HDL particle size were decreased, whereas small HDL particles were increased in T2DM (all *p* < 0.05). It is noteworthy that many of the aforementioned lipids-related alterations are present before the onset of diabetes mellitus as a result of insulin-resistant metabolic syndrome [19].

### HDL subfractions and cardiovascular risk in DM patients

Numerous large epidemiologic studies and clinical trials indicate that the mortality of CAD reasons is much higher in patients with DMT2, even after the adjustment for age, ethnicity or the presence of risk factor [98, 99]. Haffner et al. [100] found that diabetic persons with no history of myocardial infarction (MI) had equivalent rates of CAD mortality to non-diabetic individuals with a MI history. Moreover, diffuse, severe atherosclerotic lesions were observed in patients with diabetes mellitus. Potential mechanisms responsible for these worse outcomes in patients with T2D have not been fully elucidated. However, it was suggested that strict glycaemic control alone can only slightly improve diabetes-related cardiovascular events [101], Also dyslipidaemia was proposed as a causal factor of diabetic atherosclerosis as well as clinical outcomes. Also, individuals with type 1 diabetes show a considerably increased risk for cardiovascular disease in comparison to the general population [102]. Costacou et al. [24] demonstrated a smooth linear inverse association between HDL-C and CAD incidence in men. However, in DMT1 women an apparent increase in risk is observed below an HDL-C of 50 mg/dL as well as above 80 mg/dL. Moreover, their findings revealed that HDL3 subfraction was mainly associated with CAD risk. Asztalos and Schaefer [103] demonstrated deficiencies in the α1 and pre-α1–3 HDL subspecies and increase in the α3 HDL subspecies among individuals with CAD in comparison to normal controls. Their results may suggest a disturbance in the progressive increase of HDL particle size in those with CAD.

Some cross-sectional and prospective studies have suggested that HDL2 may be more protective than HDL3 [104–106]. Gordon et al. [107] revealed that young patients with T2DM exhibited decreased phospholipid content in fractions containing large HDL particles, which inversely correlated with pulse wave velocity (PWV) (*P* < 0.001). However, no relationship was observed between HDL-C and PWV. They also reported changes in 7 out of 45 identified proteins in the T2D group, including apolipoprotein (apo) A-II, apoE, and paraoxonase-1 (*p* < 0.05). Diminished ApoE content in large HDL particles may have an atherogenic effect, due to the fact that large, ApoE-rich HDL usually prevents LDL binding to proteoglycans in the vessel wall [107]. The content of ApoM which mediates the enrichment of HDL in sphingosine-1-phosphate (which promotes arterial vasodilation by stimulating endothelial nitric oxide formation [108]) was also reduced in their study. Gordon et al. [107] recommend the analysis of HDL composition, rather than HDL-C level as an useful tool in the evaluation of cardiovascular risk in this population. In patients with DMT2, reduced antioxidative properties of HDL due to the presence of hyperglycaemia and triacylglycerol enrichment has been reported [109]. In comparison to normolipidemic, non-diabetic controls, in diabetic patients specific antioxidative activity of small dense HDL3b and 3c particles was reduced up to 47% (on a particle mass or particle number basis). Moreover, plasma 8-isoprostanes were found to be considerably elevated (2.9-fold) in diabetic patients and they negatively correlated with specific antioxidative activity of HDL3 subfractions [109]. Perségol et al. [110] demonstrated the inability of HDL from type 2 diabetic patients to counteract the inhibitory effect of oxidised LDL on endothelium-dependent vasorelaxation. Results of their study suggest that HDL are less atheroprotective in type 2 diabetic patients than in control subjects. In turn, Sorrentino et al. [111] reported weaker stimulatory effect on endothelial nitric oxide synthesis in patients with type 2 diabetes. However, extended-release niacin therapy improved the capacity of HDL to stimulate endothelial nitric oxide, to decrease superoxide production, and to stimulate endothelial progenitor cell-mediated endothelial repair. Chinese prospective study of patients with stable CAD indicated that high levels of large HDL-C was inversely associated with cardiovascular risk including traditional risk factors, severity of CAD, and future cardiovascular outcomes [112]. Moreover, high large HDL-C negatively and independently correlated with the occurrence of major adverse cardiovascular events (MACEs), after adjustment for multiple confounders. The present study provided potential evidence that HDL subfraction analysis might prove useful in CAD risk assessment. However, in this study only 25% of patients had type 2 diabetes [112]. Pennathur et al. [113] study demonstrated increased levels of HDL modified by products of the myeloperoxidase system in atherosclerotic lesions and in plasma of coronary artery disease patients. Some of these modifications (i.e. chlorination) impair ABCA-1-specific cholesterol efflux [114, 115].

## Conclusions

In normal state HDL exerts various antiatherogenic properties, including reverse cholesterol transport, antioxidative and anti-inflammatory capacities. However, these properties are compromised in patients with diabetes mellitus (DM), due to oxidative modification and glycation of the HDL protein as well as the transformation of the HDL proteome into a proinflammatory protein. Numerous studies confirm that the ability of HDL to suppress inflammatory signals is significantly reduced in this group of patients. However, the exact underlying mechanisms remains to be unravelled in vivo. The understanding of pathological mechanisms underlying HDL dysfunction may enable the development of therapies targeted at specific subpopulations and focusing at the diminishing of cardiovascular risk.

## Abbreviations

ABCA1: ATP-binding cassette 1
AGEs: advanced glycation end products
ApoE: apolipoprotein E
ApoM: apolipoprotein M
CAD: cardiovascular disease
CETP: cholesteryl ester transfer protein
ChREBP: carbohydrate-responsive element-binding protein
DM: diabetes mellitus
DMT1: diabetes mellitus type 1
DMT2: diabetes mellitus type 2
GSPx-3: glutathione peroxidase 3
HDL: high-density lipoprotein
HL: hepatic lipase
IL-1: interleukin 1
LCAT: lecithin-cholesterol acyltransferase
LDL: low-density lipoprotein
Lp-PLA2: lipoprotein-associated phospholipase A2
MACEs: major adverse cardiovascular events
MCP-1: monocyte chemoattractant protein 1
PAF-AH: platelet activating factor-acetylhydrolase
PLTP: phospholipid transfer protein
PON1: paraoxonase 1
PWV: pulse wave velocity
SAA1: serum amyloid 1
SAA4: serum amyloid 4
SR-BI: scavenger receptor class B type I
SREBF1c: sterol regulatory element-binding transcription factor 1
TNF-α: tumour necrosis factor-1
